# The Effect of Foliar Putrescine Application, Ammonium Exposure, and Heat Stress on Antioxidant Compounds in Cauliflower Waste

**DOI:** 10.3390/antiox10050707

**Published:** 2021-04-29

**Authors:** Jacinta Collado-González, Maria Carmen Piñero, Ginés Otálora, Josefa López-Marín, Francisco M. del Amor

**Affiliations:** Department of Crop Production and Agri-Technology, Murcia Institute of Agri-Food Research and Development (IMIDA), C/Mayor s/n, 30150 Murcia, Spain; mariac.pinero2@carm.es (M.C.P.); gines.oralora@carm.es (G.O.); josefa.lopez38@carm.es (J.L.-M.)

**Keywords:** polyamines, cauliflower waste, heat stress, ammonium, antioxidants

## Abstract

This work has been focused on the study of how we can affect the short heat stress on the bioactive compounds content. Some recent investigations have observed that management of nitrogen fertilization can alleviate short-term heat effects on plants. Additionally, the short-term heat stress can be also ameliorated by using putrescine, a polyamine, due to its crucial role in the adaptation of plants to heat stress Therefore, different NO_3_^−^/NH_4_^+^ ratios and a foliar putrescine treatment have been used in order to increase tolerance to thermal stress in order to take advantage of the more frequent and intense heat waves and make this crop more sustainable. So, other objective of this work is to make the cauliflower waste more attractive for nutraceutical and pharmaceutical preparations. Thus, the effect of a thermal stress combined with a 50:50 NO_3_^−^/NH_4_^+^ ratio in the nutrient solution, and the foliar application of 2.5 mM putrescine increased in the content of various sugars (inositol, glucose, and fructose), total phenolic compounds and polyamines, as well as in the antioxidant activity. The greatest accumulation of these compounds was observed in young leaves. Our results show from a physiological and agronomic point of view, that the foliar application of putrescine and the 50:50 NO_3_^−^/NH_4_^+^ treatment managed to alleviate the negative effects of the abiotic stress suffered at high temperature, yielding plants with higher antioxidant compounds content.

## 1. Introduction

Globally, different types of abiotic stress drastically affect not only the productivity, but also the quality of crops [1]. It is necessary to emphasize stress due to high temperatures, because extreme temperatures, individually or combined with water-deficit conditions, represent a serious threat to agriculture, having a very negative impact on the growth, development, productivity, and quality of cultivated plants [1]. Specifically, during the 20th century, the global temperature increased by 0.5 °C and by the end of the current century it is expected to have increased by between 1.5 and 5.8 °C [1]. In recent decades, interest in the effects of climate change with regard to strengthening the intensity of abiotic stress has increased, in order to improve the abiotic stress tolerance of plants [2]. In this sense, previous works have reported that heat stress induces a set of physiological and biochemical reactions in cauliflower metabolism, activating various adaptive processes [3]. Several studies carried out in some plants of Brassicaceae family noticed that if the exposure temperature of these plants exceeds their physiological threshold (32 °C), it can lead to an increase in reactive oxygen species (ROS) within its cells, and oxidative stress may be developed when ROS exceed antioxidant defenses [3,4]. Furthermore, it is important to take in account that the oxidative stress not only depended on the intensity of heat stress suffered by plants, but also on its duration. Thus, Hussain et al. [5] in a study carried out in Chinese kale (*B. alboglabra*), it was reported that exposure to high temperature for a long time (greater than 6 days) gave rise to oxidative stress, obtaining a decrease in the content of bioactive compounds.

Spain, specifically Murcia, a region located in the southeast of Spain, is more affected by increasingly frequent heat waves [6,7]. This region is one of the regions with the highest cauliflower and broccoli production in Europe. In fact, the global cauliflower production (combined with broccoli production) amounts to 25.2 million tonnes per year [8,9]. Cauliflower (*Brassica oleracea* var. botrytis) belongs to the Brassica family. The consumption of the edible part of these vegetables has increased in recent years. This increase can be attributed to the greater interest by European consumers in consuming healthier diets. Specifically, the interest in this vegetable is that it contains a high content of fiber, minerals, vitamins, and can be considered as an excellent source of bioactive compounds that have high antioxidant activity (carotenoids, fatty acids, phenolic compounds, glucosinolates, and polyamines) [10,11,12]. Due to the wide range of phytochemicals that cauliflower contains, it may be used as preventive treatment in order to protect against many diseases, such as various types of cancers, cardiovascular disease, obesity, type II diabetes, dementia, and immune dysfunction [10,12,13]. However, the edible parts only include the inflorescence, while a larger quantity of non-edible proportion is generated. So, over 70% of this vegetable is constituted by leaves and stems. In this sense, the rise of consumption of cauliflower entails the generation of a large amount of waste each year. During its processing, a large quantity of waste is produced, the leaves constituting around 50% of the total by-product. This waste is a major problem, as it causes significant environmental effects. Therefore, in recent years, sustainable ways to reuse cauliflower waste have been studied [14,15].

In this sense, some studies have suggested that these by-products could be used as bioingredients in functional foods or as nutraceuticals. This is due to the fact that fruit and vegetable by-products including cauliflower waste could be considered as a good source of high added-value bioactive compounds. Among these bioactive compounds can be highlighted the presence of phenolic compounds and polyamines [15,16,17].

The polyamines are a group of low-molecular-weight nitrogen metabolites found in all living organisms [18]. These aliphatic amines affect the plant cell activity, and as a consequence they are involved in a wide range of biological processes that include both plant growth and development [18]. Although the mechanisms involved are still not well understood, it is known that these compounds are involved in the responses of plants to various types of abiotic stress, including heat stress [19]. In fact, several studies [5,20] reported that higher levels of free and bound polyamines in plants were obtained as consequence of the exposure of plants to heat stress. Moreover, recent studies have suggested that polyamines can be used in order to enhance the tolerance to heat stress, allowing to obtain a higher total production yield and higher quality of the final plant products [19,21]. In nature, polyamines are often found as free molecules, but they can also be found in conjugated or bound forms. Conjugated polyamines result when polyamines are linked to small molecules, such as phenolic acids, whereas when polyamines are linked to macromolecules, such as proteins, they are considered as bound polyamines. These links are due to the polycationic nature of polyamines at physiological pH. Thus, through this property, polyamines can bind to various negatively-charged sites such as those of membrane phospholipids, pectic polysaccharides, proteins, and DNA, thus being able to mediate their biological activity [22].

Among all polyamines, putrescine deserves to be highlighted, since it has been indicated that the exogenous treatment with this polyamine can promote the biosynthesis and/or accumulation of quite bioactive compounds affecting positively in the growth and development of plants. This takes place not only in inflorescence, but also in leaves of different plants. This can lead to both cauliflower more attractive by consumers and by-products cauliflower with add-value, which can allow give an additional valorization to that non-edible part of cauliflower with nutraceutical purposes [15,21]. In addition, putrescine is the precursor of other polyamines with a higher molecular weight or more nitrogen atoms in their structure, such as spermidine and spermine [21,22,23,24,25].

On the other hand, it is known that usually the plants are supplied with N in order to favor the development of the plants and to achieve a good vegetable crop production. However, caution is necessary with its use, as excessive amounts of nitrogen can have implications for human health [26]. Previous studies have reported that the form of nitrogen may be more important than the amount of total nitrogen. In fact, this has been reflected in significant effects both on the total crop production and on the quality of the final fruits and vegetables [27]. Thus, a possible solution in order to improve both the plant quality and obtaining an improved number of bioactive compounds can be vary the nutritional solution. Regarding the response of that modulation on the heat stress, some previous studies have shown that the application of NO_3_^−^/NH_4_^+^ in adequate proportions can alleviate oxidative stress caused by heat stress [21,27]. Moreover, Munene in a study carried out with amaranth species [28] indicated that a high exposure of plants to NH_4_^+^ can be reflected in a higher accumulation of all polyamines, including putrescine, cadaverine, spermidine, and spermine.

Therefore, the aim of this work was to study how to improve the resistance to heat stress of cauliflower by using of a modification of nutritive solution and the foliar application of putrescine before a short-term heat stress. Additionally, in this study also was evaluated the possibility of taking advantage of the effect of modification of nutritive solution and the foliar application of putrescine before a short-term heat stress on the cauliflower by-product quality in order to obtain cauliflower by-products with added-value for their possible use as preparations for improving human health.

For this, the effect of the exogenous application of putrescine to cauliflower leaves was studied, as well as the effects of heat and exposure to different proportions of NO_3_^−^/NH_4_^+^ in both old and young leaves, to evaluate any differences in the responses due to leaf age.

## 2. Material and Methods

### 2.1. Experimental Conditions, Plant Material and Treatments

To carry out this study, plants of the cauliflower cv. Moonshine (Enza Zaden España S.L., Almería, Spain) were used. The seeds were germinated using a mixture of peat and perlite and, after 30 days, seedlings with a similar size were selected and transplanted into 5-L black pots with coconut fiber (Pelemix, Alhama de Murcia, Spain). Previously to transplant, these pots were washed by applying 2 L of tap water. The nutrient solutions used in the experiment were applied by self-compensating drippers (2 L h^−1^). The drainage was evaluated daily in order to guarantee drainage superior to 35%. In order to evaluate the thermotolerance of the plants as a function of the applied nitrogen (N) source and the application of putrescine, the experiments were carried out in a climatic chamber designed by del Amor et al. [29], with fully-controlled environmental conditions: 60% relative humidity, 16/8 h day/night with 28/16 °C at first and 43/30 °C during the heat stress and an ambient CO_2_ concentration of 400 µmol mol^−1^ CO_2_. The photosynthetically active radiation (PAR) of 250 µmol m^−2^ s^−1^ was provided by a combination of fluorescent lamps (TL-D Master reflex 830 and 840, Koninklijke Philips Electronics N.V., The Netherlands) and high-pressure lamps (Son-T Agro, Philips).

The N treatments consisted of three solutions with different proportions of NO_3_^−^/NH_4_^+^ (100/0, 80/20, and 50/50). Twenty plants per treatment were used. The experiment started under the initial thermal conditions and on day 86 half of the plants, which were randomly selected, received a foliar spraying with a solution containing 2.5 mM putrescine plus 0.01% Tween-20 as a surfactant. Each plant was fully sprayed with 20 mL of the putrescine solution, using a hand sprayer. The putrescine treatment was allowed to act for four days and then half of the sprayed plants and half of the non-sprayed plants were harvested. The plants remaining in the climatic chamber were then exposed to a heat stress at 43/30 °C day/night for three days. After that, they were harvested. The experiment ended 93 days after transplantation.

### 2.2. Chemicals and Reagents

Sodium hydroxide and SPE cartridges (C18 Sep-Pak cartridges) were acquired from Fluka (Buchs, Switzerland) and Waters Associates (Milford, Mass.), respectively. Sugars (glucose, sucrose, fructose, and inositol), polyamines (spermidine, spermine, cadaverine, histamine, putrescine and 1,6-hexaendiamine), gallic acid, 6-hydroxy-2,5,7,8-tetramethylchroman-2-carboxylic acid (Trolox), 2,2-azino-bis(3-ethylbenzothiazoline-6-sulphonic acid) diammonium salt (ABTS^•+^) and benzoyl chloride were purchased from Sigma-Aldrich (Steinheim, Germany). Methanol, acetonitrile (LC-MS grade), sodium carbonate, Folin–Ciocalteu reagent and ethyl ether were obtained from Panreac Química (Barcelona, Spain). Ultrapure water was produced using a Millipore water purification system.

### 2.3. Growth and Shoot and Leaf Weights

The height of the cauliflower plants was determined weekly. The shoot and leaf weights were determined on the same days and for the same plants where the photosynthetic parameters had previously been measured. For the determination of the shoot and leaf weights, intact plants were placed in a collection tray with the shoot and roots in separate compartments. After weighing the whole shoot, the outer and young leaves were separated and weighed.

### 2.4. Determination of the Total Phenolic Compounds and Antioxidant Activity (ABTS^●^^+^)

The determination of the total phenolic compounds (TPC) and antioxidant activity was performed in both young and outer cauliflower leaves. The determination of phenolic compounds from fresh leaves was carried out using the Folin–Ciocâlteu colorimetric method [30]. For this, a 0.5 g sample was homogenized with 5 mL of extractant (acetone, 80%) and centrifuged at 10,000× *g*, at 4 °C, for 10 min. Later, 100 µL of the supernatant were mixed with 1 mL of Folin–Ciocâlteu reagent (diluted with Milli-Q water, 1:10) and 2 mL of Milli-Q water. This mixture was incubated at room temperature for 3 min and then 5 mL of 20% sodium carbonate were added, followed by a re-incubation in the dark at room temperature for 1 h. Then, the absorbance of the blue-colored mixture was measured at 765 nm using a UV–visible spectrophotometer (Shimadzu UV-1800 model with the CPS-240 cell holder, Shimadzu Europa GmbH, Duisburg, Germany). The results were calculated according to a calibration curve of gallic acid and were expressed as gallic acid equivalents (GAE), µg GAE g^−1^. For all treatment combinations, five replicates were analysed.

The antioxidant activity was measured in freeze-dried young and outer cauliflower leaves using the ABTS^●^^+^ radical, as previously reported [31]. The method of extraction consisted of mixing 0.5 g of sample with 10 mL of extractant (MeOH/water (80:20, *v/v*) + 1% HCl) at room temperature, followed by sonication for 15 min and storage for 24 h at 4 °C. The resultant extract was sonicated for 15 min and centrifuged for 10 min at 10,000× *g*. A 10 μL sample of the supernatant was mixed with 990 µL of the ABTS^●^^+^ (2,2-azinobis-(3-ethylbenzothiazoline-6-sulphonic acid)) radical cation solution, shaken and placed in darkness for 10 min. Then, the absorbance was measured at a wavelength of 734 nm using a UV–visible spectrophotometer (Shimadzu CPS-240 model, Kyoto, Japan). The antioxidant activity was quantified according to a Trolox calibration curve (0.01–3 mmol Trolox L^−1^) and expressed in µmol Trolox g^−1^.

### 2.5. Extraction and Quantification of Total Soluble Sugars

Soluble sugars were extracted as described by Balibrea et al. [32], with some modifications. Briefly, 50 mg of lyophilized young and outer leaves were incubated twice with 1.5 mL of 80% methanol (*v/v*), at 4 °C, for 30 min each time. After centrifugation for 15 min at 3500× *g*, at 4 °C, each supernatant was filtered through a C18 Sep-Pak cartridge (Waters Associates, Milford, MA), which had been activated previously with 20 mL of methanol/water (80%/20%). The two supernatants were combined and filtered through a 0.45 μm filter (Millipore, Beford, MA, USA). The concentrations of inositol, glucose, fructose, and sucrose in the extracts were determined directly by ion chromatography with an 817 Bioscan (Metrohm, Herisau, Switzerland) system equipped with a pulsed amperometric detector (PAD) and a gold electrode, using a METROHM Metrosep Carb 1-150 IC column (4.6 × 250 mm), which was heated to 32 °C.

### 2.6. Extraction of Polyamines and Their Analysis by UHPLC

Polyamines from 5 g of fresh young and outer leaves were extracted by homogenization with 1.2 mL of 5% (*v/v*) cold HClO_4_ for 1 min, using an ultraturrax (Ika, Staufen, Germany). The extract was centrifuged (Eppendorf centrifuge 5804R, Hamburg, Germany) at 12,000× *g* for 8 min, at 4 °C. The supernatants were derivatized with benzoyl chloride, using a slight modification of the technique used by Rodríguez et al. [33]. An aliquot of 500 µL of supernatant was mixed with 2 mL of 2 M NaOH and 20 µL of benzoyl chloride; this mixture was vortexed for 15 s and incubated for 20 min at room temperature. Then, 4 mL of a saturated NaCl solution were added in order to stop the reaction and the polyamines were extracted with 4 mL of cold diethyl ether. Both resulting phases (aqueous and organic) were stored at −20 °C until the extraction of the total polyamines in the ether phase had been achieved. From the resulting upper phase, 1.5 mL were evaporated by a SpeedVac Evaporator (Savant SPD121P, Thermo Scientific, Waltham, MA, USA). The resulting sample was dissolved in 500 µL of the mobile phase (water/acetonitrile, 58/42%, *v/v*). Standards of polyamines were treated in a similar way. Aliquots of 10 µL were injected into a UHPLC-DAD (Waters, Milford, USA). To obtain a satisfactory chromatographic separation, an ACQUITY UPLC HSS T3 column (2.1 × 100 mm, 1.8 µm) (Waters Corp., Wexford, Ireland) was used with only one eluent: water plus acetonitrile (58/42%, *v/v*). The elution was performed at a flow rate of 0.55 mL min^−1^ and the column temperature was kept at 40 °C. Data acquisition and processing were performed using Empower 2 (Waters) software. Polyamine peaks were detected at 254 nm. Five replicates per treatment were analysed.

### 2.7. Statistical Analysis

The experiment had a completely randomized design with a 3 × 2 × 2 factorial arrangement composed of three nitrogen sources ratio (control (100:0), 80:20 and 50:50), two putrescine application (absence and presence of putrescine at 2.5 mM), and two temperature levels (28 °C and 43 °C). Analysis were performed six repetition per treatment (and individual plant per plot was considered one repetition). The data was tested first for homogeneity of variance and normality of distribution. To check the regression model hypothesis (linearity, homoscedasticity, normality, and independency), the Kolmogorov–Smirnov test was used with the Liliefors correction and the Shapiro–Wilk test for normality and the Levene test for homoscedasticity on the typified residuals. An analysis of variance (ANOVA) was performed, considering the different treatments, when the data were in accordance with the assumptions of normality and homogeneity of variance. To determine differences among the mean values, a post hoc Tukey’s test (*p* ≤ 0.05) in SPSS v.21 (IBM, Chicago, IL, USA) was conducted. Values for each replicate were averaged before the mean and standard error (SE) of each treatment were calculated. Combinations of treatments were used—involving three NO_3_^−^/NH_4_^+^ ratios (control (100:0), 80:20 and 50:50), two temperatures (28 °C and 43 °C) and the presence/absence of 2.5 mM putrescine—with five plants per combination.

## 3. Results and Discussion

### 3.1. Analysis of Biomass

**Figure 1:**
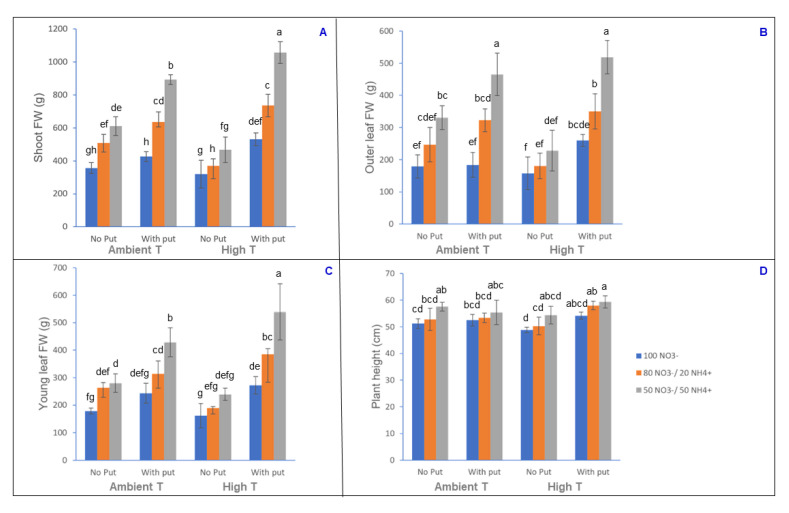
Effect of heat stress, different NO_3_^−^/NH_4_^+^ ratios and the foliar application of 2.5 mM putrescine on shoot FW (**A**), outer leaf FW (**B**), young leaf FW (**C**), and plant height (**D**) of cauliflower. For data analysis, an ANOVA was performed and the pairwise changes were defined using the Tukey post hoc test. Thus, different letters represent significantly different mean values according to the Tukey test at *p* ≤ 0.05.

Under heat stress, and with increased NH_4_^+^ in the nutrient solution, the cauliflower plants had greater biomass than in control conditions (Figure 1A–D). A similar trend was found by other authors for plants exposed to several abiotic stresses and a nutrient solution enriched with NH_4_^+^ [34,35,36,37]. Previous work has shown higher tolerance of abiotic stresses in different plant species according to the NO_3_^−^/NH_4_^+^ ratio [34]. Some studies claimed that the foliar application of putrescine at an adequate concentration, as in our case, can give rise to a series of physiological processes in the plant and generate the biosynthesis of bioactive compounds, some of them being osmoprotective. This response is in order to alleviate/compensate the negative effects on the plant biomass of the abiotic stress. In this way, the foliar application of putrescine can give rise not only to a plant of higher quality, but also a healthier plant [38]. Of note is the fact that the metabolism of polyamines is related not only to the generation of reactive oxygen species, but also the production of nitric oxide. This gaseous molecule not only acts as an intra- and intercellular messenger, but also as an intermediate signalling molecule for plant growth [39]. Yang et al., [40] reported that exogenous application of putrescine induced nitric oxide generation in soybean. This could be a consequence of the fact that polyamines can modulate the arginine-linked nitric acid synthase and nitrate reductase pathways [38]. Thus, according to several authors, this positions this molecule as an intermediary involved in various physiological effects and the mitigation of stress [38].

### 3.2. Evaluation of the Antioxidant Activity and Total Phenolic Compounds

**Figure 2:**
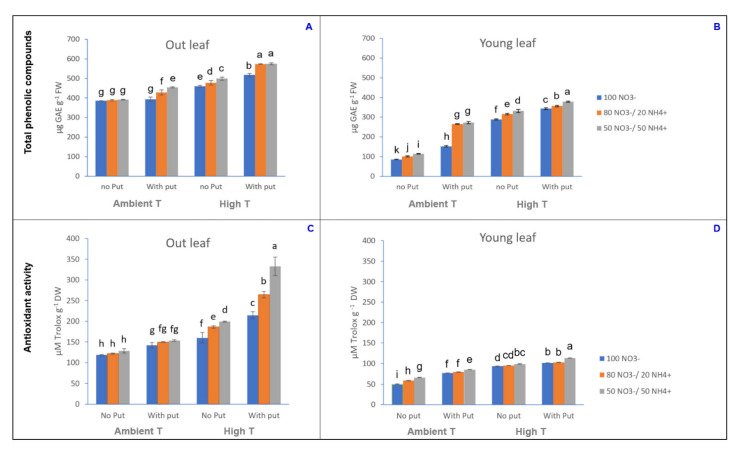
Total phenolic compounds in outer and young leaves (**A**,**B**), respectively and antioxidant activity in outer and young leaves (**C**,**D**), respectively of the cauliflower cv. Moonshine. For data analysis, an ANOVA was performed and the pairwise changes were defined using the Tukey post hoc test. Thus, different letters represent significantly different mean values according to the Tukey test at *p* ≤ 0.05.

The extracts from cauliflower waste seem to be a good source of TPC and they showed good scavenging activity against ABTS^•+^ radicals. Our results show that TPC content varied from 80.5 µg GAE g^−1^ FW, in the outer leaves under the control treatment, to 574.8 µg GAE g^−1^ FW, in the younger leaves of plants subjected to a short heat shock and grown with a 50:50 NO_3_^−^/NH_4_^+^ ratio (Figure 2). Taking into consideration that in our experiment the moisture content of the cauliflower leaves varied between 9% (in younger leaves subjected to heat stress, a 50:50 NO_3_^−^/NH_4_^+^ ratio and putrescine application) and 30% (in outer leaves in the control treatment) (data not shown), the TPC content ranged between 268.33 µg GAE g^−1^ DW and 6386.7 µg GAE g^−1^ DW. Although these values are lower than those found in the edible part of the white cauliflower cv. Moonshine [41], they can be considered to be in accordance with the TPC concentrations reported by other authors for cauliflower waste: between 4855.8 µg GAE g^−1^ DW and 6109 µg GAE g^−1^ DW [42]. Figure 2 shows that the outer and young leaves had antioxidant activity values between 49.00 and 332.6 µmol Trolox g^−1^ DW, in agreement with those found by other authors for extracts of raw cauliflower by-products [15] and intermediate in comparison with other leafy species of the Brassicaceae [43]. Some previous works have attributed such high antioxidant activity not only to phenolic compounds, but also to substances such as soluble fiber, glucosinolates, and their derived products, such as isothiocyanates, that are present in cauliflower waste and have potent anticancer effects [15].

Although other authors have reported the possibility of using cauliflower residues from various points of view [14,15], this is the first work where their beneficial properties have been studied in order to be improved with regard to the pharmaceutical, cosmetics, and food industries. It is known that thermal stress is accompanied by an increase in the concentration of reactive oxygen species [4]. In this study, the short-term heat stress had a significant impact on the TPC and antioxidant activity of cauliflower leaves (Figure 2, Table 1, Table S1). This increase in TPC and antioxidant activity can be ascribed to a higher PAL (phenylalanine ammonia-lyase) activity resulting from thermal stress, since PAL is considered one of the main lines of cell acclimation against stress in plants [44].

The nutrient solution NO_3_^−^/NH_4_^+^ ratio also had a significant effect on the TPC and antioxidant activity of cauliflower leaves (Figure 2). It is known that N fertilization practices have an important influence on the biosynthesis of phenolic compounds. In fact, previous findings showed that the total phenolics content is significantly increased in N-starved plants. This indicates that the biosynthesis of secondary plant metabolites is stimulated by a lower availability of N, inducing a plant defense mechanism against nutritional stress. It can be ascribed to the fact that the accumulation of excess carbon in plants in response to nutrient stress leads to higher production of secondary carbon-based metabolites. In line with this hypothesis, a study carried out with Carica papaya revealed that phenolic compounds act as primary antioxidants [28]. In several studies, plants exposed to a high concentration of NH_4_^+^ showed a high accumulation of polyamines, which act as precursors of some secondary metabolites, alleviating the stress [28].

The phenolic compounds and antioxidant activity in cauliflower leaves were increased as a result of the exogenous application of putrescine, as well. This is in agreement with Chen and collaborators [38], who indicated that polyamines promote not only photosynthetic activity, but also the antioxidant capacity and osmotic adjustment ability of plants. In particular, in work carried out in wheat, foliar treatment with putrescine (2.5 mM) alleviated the adverse effect of high temperature, increasing the thermotolerance [45]. As can be seen in Figure 2, in the present study, the distribution of phenolic compounds varied as a consequence of the application of thermal and nutritional stresses, and the content of phenolic compounds also varied with the age of the leaves. In this sense, our data show that the young leaves from stressed plants had a greater accumulation of TPC than the outer leaves. This finding is in accordance with several studies performed in many higher plant species during the last two decades, which stated that young tissues generally have higher accumulation of secondary metabolites overall in plants that suffer any abiotic stress [44].

### 3.3. Determination of Sugars

The chromatographic profile of the sugars in the cauliflower leaves was constituted by four free sugars (inositol, glucose, fructose, and sucrose). These sugars were determined by comparison of their retention times with those of commercial standards. As can be seen in Table 2 (and Table S2) the total sugars content oscillated between 159.57 and 251.20 mg kg^−1^ DW for younger leaves, and for outer leaves between 241.04 and 313.99 mg kg^−1^ DW. In the case of the individual free sugars, glucose exhibited the highest content (*p* ≤ 0.05), whilst sucrose was the least abundant. The levels of total free sugars in the freeze-dried cauliflower leaves in the current work were higher than those found by Bhandari and Kwak [42], who reported values of 192.0, 214.8, and 224.4 mg g^−1^ for cauliflower leaves, depending on the cultivar. As expected, due to their role as osmoprotective metabolites, the content of these free sugars was positively influenced by the short heat stress [46,47]. This may be ascribed to inhibition of sucrose synthase or invertase activity, so that during stress sucrose is cleaved into fructose and glucose [46,47]. A similar response to heat stress, to achieve an adequate osmotic adjustment, was obtained when the effect of the nutrient solution NO_3_^−^/NH_4_^+^ ratio on the sugars content was studied. Thus, the results displayed in Table 3 (and Table S3) show that the sugar concentration in cauliflower leaves was positively influenced by an increase in the proportion of NH_4_^+^ to 20% or 50% in the nutrient solution. These results are consistent with those obtained previously, where it was observed that those plants nourished with higher proportions of NH_4_^+^ could exhibit higher energy efficiency [27]. Of note is the fact that, in our study, the increase in the total sugars content was more pronounced when the NO_3_^−^/NH_4_^+^ ratio supplied to plants subjected to high temperatures was altered. The highest and lowest concentration of sucrose and fructose, respectively, was observed in plants grown with the 50:50 NO_3_^−^/NH_4_^+^ ratio, while the highest concentration of glucose was observed with the 80:20 NO_3_^−^/NH_4_^+^ ratio. These findings are in accordance with those reported by Petropoulos et al. [27], who found that an increase in sucrose at a NO_3_^−^/NH_4_^+^ ratio of 50:50 may have been due more to fructose than to glucose.

Table 2 and Table 3 show that the spraying of the cauliflower leaves with putrescine increased the accumulation of sugars. This may be due to the counteracting of increased reactive oxygen species levels by the sprayed polyamine and, in turn, to the direct correlation that exists between the reactive oxygen species present in the cells and the sugars accumulated. So, in addition to acting as osmoprotective metabolites, sugars could act as reactive oxygen species scavengers and help to achieve membrane stabilization under abiotic stress [39]. Thus, our results suggest that the effect of putrescine (2.5 mM) foliar treatments on cauliflower plants may be related to their thermotolerance through enhanced osmoregulation.

As happened with the TPC, the sugars content was higher in young leaves than in outer ones. This finding is in agreement with previous results showing that young leaves have high metabolic rates and a great demand for resources, and thus compete with other organs of the plant [39].

### 3.4. Identification and Quantification of Polyamines by UHPLC-DAD

In this work the cauliflower leaves contained four free polyamines (putrescine, cadaverine, spermidine, and spermine). They were identified and tentatively quantified by taking into account their retention times and by comparison with the peaks of commercial standards. Table 4 and Table 5 show that the polyamines contents varied significantly, with values between 20.49 and 63.16 nmols g^−1^ for outer leaves and between 51.84 and 97.05 nmols g^−1^ for younger leaves. Putrescine was the most abundant, followed by spermidine, and the least abundant was spermine (Table 4 and Table 5). The contents are high with respect to those obtained for leaves of *Brassica napus* [44].

To our knowledge, although quite a lot of work has been published about the effects of polyamines on plants under salinity and drought stresses, there has been little work focused on polyamines in plants under heat stress. In addition, there are several different points of view on the relationship between polyamines and plant heat stress [47]. In this sense, several authors reported that an increase in spermidine and a decrease in putrescine were obtained as a consequence of the exposure of plants to heat stress [44]. However, in our work, the contents of all polyamines increased as a result of a short-term exposure to high temperature. These results are consistent with a report that endogenous leaf polyamines in Chinese kale were increased after 6 days of high-temperature treatment [5]. Polyamines perform a large number of functions in plants, including promotion of photosynthesis and increasing the antioxidant capacity and osmotic adjustment ability (as already indicated in the sugars) in order to reduce the damage caused by different types of stress. The controversy regarding the accumulation of these metabolites must be due to the fact that the main physiological mechanisms of tolerance of high temperatures differ among plant species and among the stages of plant maturation [38]. The results in Table 4 and Table 5 show that, as happened with the TPC, polyamines accumulated in those cauliflower plants whose nutrient solution was more enriched with ammonium. This is supported by several authors who proposed that the content of polyamines is raised to high levels by ammonium nutrition in order to enhance plant tolerance of stresses [28]. Although the roles of polyamines have been assigned mainly to floral induction, reproductive processes and root formation, a growing body of evidence points to an important role in the management of nitrogen stresses. For this, polyamines act as nitrogen reserves or deposits and as free radical scavengers in order to maintain the integrity of the membranes and, consequently, protect cells from nitrogen toxicity [28].

The exogenous polyamine treatment increased the endogenous polyamine content. This is consistent with other studies that reported that exogenous polyamine application can increase the polyamines content in plants, which can lead to a decline in reactive oxygen species and thus in the negative effects of abiotic stress, improving plant quality and even delaying senescence [38].

From a nutritional point of view, although in cauliflower waste no new compounds were found compared to the edible part, the non-edible part is a good and cheap source of some bioactive compounds—phenolics, sugars, and polyamines, among others. Some nutritional studies have indicated that there is a relationship between polyamine-rich foods and a lower risk of cardiovascular diseases, better conservation of memory and greater longevity [11]. Therefore, it could be interesting to apply this new strategy (the combination of a short heat stress, a nutrient solution with a 50:50 NO_3_^−^/NH_4_^+^ ratio and foliar application of putrescine) to promote the incorporation of these cauliflower by-products into nutraceutical and pharmaceutical preparations for human health.

## 4. Conclusions

Based on all these results, we can conclude that the use of putrescine and an ammonium ratio of 50% at a high temperature gave rise to plants with greater biomass and a higher content of bioactive compounds. The young leaves showed the greatest biomass and were the site where the biosynthesis of interesting bioactive compounds was mainly promoted. The stimulation of polyamines accumulation in the cauliflower cv. Moonshine by the use of the combination of a short heat stress, a nutrient solution with a 50:50 NO_3_^−^/NH_4_^+^ ratio, and foliar application of putrescine can transform its waste into a very rich source of polyamines. Consequently, the development of this new strategy in cauliflower to obtain improved wastes has promising prospects for the pharmaceutical, cosmetics and food industries. The processing of these wastes would have another advantage since their environmental impact would be greatly reduced.

## Figures and Tables

**Table 1:** Explanation statistical of the data exhibited in Figure 2.

Main Effects	Total Phenolic Compounds		Antioxidant Activity	
	Outer leaves	Young leaves	Outer leaves	Young leaves
T	***	***	***	***
Put	***	***	***	***
NO_3_^−^/NH_4_^+^	***	***	***	***
T X Put	ns	ns	***	*
T X NO_3_^−^/NH_4_^+^	***	**	***	ns
Put X NO_3_^−^/NH_4_^+^	***	***	***	***
T X Put X NO_3_^−^/NH_4_^+^	***	***	***	***

The effect of heat stress, different NO_3_^−^/NH_4_^+^ ratios and the foliar application of 2.5 mM putrescine. The data are presented as the treatment means (*n* = 5). Different letters represent significantly different mean values according to the Tukey test at *p* ≤ 0.05. Analysis of variance: ns, not significant; * *p* ≤ 0.05; ** *p* ≤ 0.005; *** *p* ≤ 0.001.

**Table 2:** Effect of the foliar application of putrescine on the concentrations of sugars (g kg^−1^ DW) in outer leaves of the cauliflower cv. Moonshine at different NO_3_^−^/NH_4_^+^ ratios and temperatures.

Temperature	NO_3_^−^/NH_4_^+^		Inositol	Glucose	Fructose	Sucrose	Total Free Sugars
Ambient temperature	100/0	Without Put	5.50 h	39.71 g	12.91 g	101.43 f	159.57 c
		With Put	11.04 f	71.12 e	18.34 e	108.03 e	208.54 b
	80/20	Without Put	8.08 g	75.80 e	11.31 h	114.33 d	209.53 ab
		With Put	12.38 f	85.41 cd	15.84 f	123.22 c	236.86 a
	50/50	Without Put	8.75 g	60.47 f	9.25 i	142.90 b	221.38 ab
		With Put	15.02 e	83.08 de	13.84 g	149.75 a	261.70 a
High temperature	100/0	Without Put	18.69 d	97.25 bc	25.49 b	72.78 j	214.22 ab
		With Put	21.80 c	101.32 ab	28.14 a	82.37 i	233.64 a
	80/20	Without Put	20.34 cd	105.92 a	23.49 c	76.35 j	226.12 ab
		With Put	23.37 b	112.18 a	27.76 a	88.39 h	251.71 a
	50/50	Without Put	21.46 c	93.97 bc	21.06 d	95.29 g	231.79 ab
		With Put	25.34 a	99.71 ab	27.38 a	98.75 fg	251.20 a
**Main effects**							
Temperature (T)			***	***	***	***	***
Putrescine (Put)			***	***	***	***	*
Nitrate/ammonium (NO_3_^−^/NH_4_^+^)			***	***	***	***	***
T X Put			ns	***	ns	ns	*
T X NO_3_^−^/NH_4_^+^			ns	***	ns	**	***
Put X NO_3_^−^/NH_4_^+^			***	***	***	***	ns
T X Put X NO_3_^−^/NH_4_^+^			*	***	**	***	**

Different small letters within a column indicate significant differences among the different putrescine treatments, NO_3_^−^/NH_4_^+^ ratios and temperatures (*p* = 0.05, Tukey test). Analysis of variance: ns, not significant; * *p* ≤ 0.05; ** *p* ≤ 0.005; *** *p* ≤ 0.001.

**Table 3:** Effect of the foliar application of putrescine on the concentrations of sugars (g kg^−1^ DW) in younger leaves of the cauliflower cv. Moonshine at different NO_3_^−^/NH_4_^+^ ratios and temperatures.

Temperature	NO_3_^−^/NH_4_^+^		Inositol	Glucose	Fructose	Sucrose	Total free sugars
Ambient temperature	100/0	Without Put	26.53 i	117.08 i	31.75 fg	65.68 bc	241.04 g
		With Put	30.39 fg	137.29 fg	36.12 cd	70.81 a	274.62 e
	80/20	Without Put	28.07 hi	128.75 gh	31.67 fg	67.43 b	256.00 f
		With Put	31.61 ef	152.33 e	34.25 de	71.49 a	289.68 bcd
	50/50	Without Put	28.64 gh	122.76 hi	30.47 h	69.95 a	251.82 fg
		With Put	32.62 de	143.41 f	32.67 ef	72.52 a	281.22 cde
High temperature	100/0	Without Put	34.28 cd	153.88 de	36.98 bc	51.04 g	276.18 de
		With Put	37.52 b	158.78 cde	40.76 a	59.36 e	302.45 b
	80/20	Without Put	35.97 bc	165.02 bc	36.82 bc	56.53 f	292.29 bc
		With Put	37.30 b	175.32 a	38.83 b	61.51 de	312.99 a
	50/50	Without Put	37.32 b	162.96 cd	36.05 cd	59.54 e	291.90 bc
		With Put	39.82 a	172.64 ab	38.14 bc	63.38 cd	313.99 a
**Main effects**							
Temperature (T)			***	***	***	***	***
Putrescine (Put)			***	*	**	***	**
Nitrate/ammonium (NO_3_^−^/NH_4_^+^)			***	***	***	***	***
T X Put			ns	ns	ns	ns	ns
T X NO_3_^−^/NH_4_^+^			ns	ns	ns	*	ns
Put X NO_3_^−^/NH_4_^+^			***	***	***	***	***
T X Put X NO_3_^−^/NH_4_^+^			ns	*	ns	ns	ns

Different small letters within a column indicate significant differences among the different putrescine treatments, NO_3_^−^/NH_4_^+^ ratios and temperatures (*p* = 0.05, Tukey test). Analysis of variance: ns, not significant; * *p* ≤ 0.05; ** *p* ≤ 0.005; *** *p* ≤ 0.001.

**Table 4:** Effect of the foliar application of putrescine on the concentrations of polyamines (nmols g^−1^) in outer leaves of the cauliflower cv. Moonshine at different NO_3_^−^/NH_4_^+^ ratios and temperatures.

Temperature	NO_3_^−^/NH_4_^+^		Putrescine	Cadaverine	Spermidine	Spermine	Total
Ambient temperature	100/0	Without Put	6.30 j	7.33 j	4.50 k	2.37 c	20.49 k
		With Put	13.74 g	9.10 h	10.63 h	3.22 abc	36.69 h
	80/20	Without Put	8.24 i	8.49 i	5.85 j	2.82 bc	25.41 j
		With Put	15.62 f	10.03 g	12.34 g	3.07 abc	41.04 g
	50/50	Without Put	11.36 h	11.09 f	8.66 i	2.93 abc	34.04 i
		With Put	16.45 e	12.37 d	13.59 f	3.31 abc	45.72 f
High temperature	100/0	Without Put	17.21 e	11.76 e	14.47 e	3.37 abc	46.81 e
		With Put	21.65 b	14.99 b	16.82 c	3.45 ab	56.91 c
	80/20	Without Put	18.14 d	13.31 c	15.55 d	3.53 ab	50.53 d
		With Put	22.34 a	15.29 ab	19.51 b	3.73 ab	60.87 b
	50/50	Without Put	20.76 c	14.87 b	16.14 cd	3.60 ab	55.37 c
		With Put	22.74 a	15.63 a	20.84 a	3.95 a	63.16 a
**Main effects**							
Temperature (T)			***	***	***	***	***
Putrescine (Put)			***	***	***	*	***
Nitrate/ammonium (NO_3_^−^/NH_4_^+^)			***	***	***	ns	***
T X Put			***	***	*	ns	***
T X NO_3_^−^/NH_4_^+^			***	**	**	ns	***
Put X NO_3_^−^/NH_4_^+^			***	***	***	ns	***
T X Put X NO_3_^−^/NH_4_^+^			***	***	***	***	***

Different small letters within a column indicate significant differences among the different putrescine treatments, NO_3_^−^/NH_4_^+^ ratios and temperatures (*p* = 0.05, Tukey test). Analysis of variance: ns, not significant; * *p* ≤ 0.05; ** *p* ≤ 0.005; *** *p* ≤ 0.001.

**Table 5:** Effect of the foliar application of putrescine on the concentrations of polyamines (nmoles g^−1^) in younger leaves of the cauliflower cv. Moonshine at different NO_3_^−^/NH_4_^+^ ratios and temperatures.

Temperature	NO_3_^−^/NH_4_^+^		Putrescine	Cadaverine	Spermidine	Spermine	Total
Ambient temperature	100/0	Without Put	23.12 h	12.44 d	17.72 h	3.20 h	51.84 i
		With Put	24.17 g	16.14 bc	27.66 efg	4.45 fg	68.63 h
	80/20	Without Put	23.73 g	15.93 c	24.80 g	4.17 g	71.02 h
		With Put	25.15 f	16.68 abc	29.61 def	4.54 fg	72.41 gh
	50/50	Without Put	24.016 g	16.12 bc	26.60 fg	4.29 fg	75.98 fgh
		With Put	26.33 e	17.03 abc	30.78 de	4.77 def	78.91 efg
High temperature	100/0	Without Put	27.00 d	17.86 abc	31.73 cd	5.01 cde	81.59 def
		With Put	29.73 b	18.71 abc	37.57 ab	5.42 abcd	84.60 cde
	80/20	Without Put	27.63 c	18.17 abc	33.44 cd	5.37 bcd	86.97 bcd
		With Put	29.90 b	19.05 ab	39.064 ab	5.94 ab	91.62 abc
	50/50	Without Put	28.19 c	18.19 abc	35.19 bc	5.61 abc	93.95 ab
		With Put	30.53 a	19.44 a	40.96 a	6.11 a	97.05 a
**Main effects**							
Temperature (T)			***	***	***	***	***
Putrescine (Put)			***	ns	***	*	***
Nitrate/ ammonium (NO_3_^−^/NH_4_^+^)			***	ns	***	***	***
T X Put			ns	ns	ns	ns	ns
T X NO_3_^−^/NH_4_^+^			ns	ns	ns	ns	ns
Put X NO_3_^−^/NH_4_^+^			***	ns	***	***	***
T X Put X NO_3_^−^/NH_4_^+^			***	ns	ns	ns	ns

Different small letters within a column indicate significant differences among the different putrescine treatments, NO_3_^−^/NH_4_^+^ ratios and temperatures (*p* = 0.05, Tukey test). Analysis of variance: ns, not significant; * *p* ≤ 0.05; *** *p* ≤ 0.001.

## Data Availability

Data is contained within the article and supplementary material.

## Supplementary Materials

The supplementary materials are available at https://www.mdpi.com/article/10.3390/antiox10050707/s1.
