# Supplementary material for: The Effect of Foliar Putrescine Application, Ammonium Exposure, and Heat Stress on Antioxidant Compounds in Cauliflower Waste
Source: Antioxidants (Basel). 2021 Apr 29;10(5):707. doi: 10.3390/antiox10050707 (PMC8147109; doi:10.3390/antiox10050707)
Supplement: Supplementary file 1 [file antioxidants-10-00707-s001.zip › antioxidants-1178824-supplementary.pdf]

Supplemental Table 1. p-Value of statistical analysis of figure 2.				
Main effects	Total phenolic compounds		Antioxidant activity	
	Outer leaves	Young leaves	Outer leaves	Young leaves
T	0.000	0.000	0.000	0.000
Put	0.000	0.000	0.000	0.000
NO_3^-/ NH_4^+	0.000	0.000	0.000	0.000
T X Put	0.590	0.703	0.000	0.051
T X NO_3^-/ NH_4^+	0.000	0.003	0.000	0.055
Put X NO_3^-/ NH_4^+	0.000	0.000	0.000	0.000
T X Put X NO_3^-/ NH_4^+	0.000	0.000	0.000	0.000

Supplemental Table 2. p-Value of statistical analysis of Table 2. Effect of the foliar application of putrescine on the concentrations of sugars (g kg^{-1} DW) in outer leaves of the cauliflower cv. Moonshine at different NO_3^-/NH_4^+ ratios and temperatures.

Temperature	NO_3^-/NH_4^+	Inositol	Glucose	Fructose	Sucrose	Total free sugars
**Main effects**						
Temperature (T)		0.000	0.000	0.000	0.000	0.000
Putrescine (Put)		0.000	0.000	0.000	0.000	0.007
Nitrate/ammonium (NO_3^-/NH_4^+)		0.000	0.000	0.000	0.000	0.000
T X Put		0.063	0.000	0.653	0.185	0.028
T X NO_3^-/NH_4^+		0.867	0.000	0.127	0.003	0.000
Put X NO_3^-/NH_4^+		0.000	0.000	0.000	0.000	0.255
T X Put X NO_3^-/NH_4^+		0.18	0.000	0.010	0.000	0.021

Supplemental Table 2 continued. p-Value of statistical analysis of Table 2 continued.						
**Temperature**	**NO_3^-/NH_4^+**	**Inositol**	**Glucose**	**Fructose**	**Sucrose**	**Total free sugars**
**Main effects**						
Temperature (T)		0.000	0.000	0.000	0.000	0.000
Putrescine (Put)		0.000	0.008	0.002	0.000	0.002
Nitrate/ammonium (NO_3^-/NH_4^+)		0.000	0.000	0.000	0.000	0.000
T X Put		0.886	0.664	0.953	0.244	0.926
T X NO_3^-/NH_4^+		0.424	0.593	0.593	0.026	0.609
Put X NO_3^-/NH_4^+		0.000	0.000	0.000	0.000	0.000
T X Put X NO_3^-/NH_4^+		0.108	0.048	0.824	0.254	0.241

Supplemental Table 3. p-Value of statistical analysis of Table 3.						
**Temperature**	**NO_3^-/NH_4^+**	**Putrescine**	**Cadaverine**	**Spermidine**	**Spermine**	**Total**
**Main effects**						
Temperature (T)		0.000	0.000	0.000	0.000	0.000
Putrescine (Put)		0.000	0.000	0.000	0.019	0.000
Nitrate/ammonium (NO_3^-/NH_4^+)		0.000	0.000	0.000	0.137	0.000
T X Put		0.000	0.000	0.042	0.732s	0.000
T X NO_3^-/NH_4^+		0.000	0.002	0.002	0.201	0.000
Put X NO_3^-/NH_4^+		0.000	0.000	0.000	0.174	0.000
T X Put X NO_3^-/NH_4^+		0.000	0.000	0.000	0.000	0.000

Supplemental Table 3 continued. p-Value of statistical analysis of Table 3 continued.						
**Temperature**	**NO_3^-/NH_4^+**	**Putrescine**	**Cadaverine**	**Spermidine**	**Spermine**	**Total**
**Main effects**						
Temperature (T)		0.000	0.000	0.000	0.000	0.000
Putrescine (Put)		0.000	0.111	0.000	0.013	0.000
Nitrate/ ammonium (NO_3^-/NH_4^+)		0.000	0.072	0.000	0.000	0.000
T X Put		0.205	0.403	0.465	0.445	0.506
T X NO_3^-/NH_4^+		0.142	0.309	0.234	0.742	0.243
Put X NO_3^-/NH_4^+		0.000	0.062	0.000	0.000	0.000
T X Put X NO_3^-/NH_4^+		0.000	0.412	0.442	0.442	0.515
